# Englerin A induces an acute inflammatory response and reveals lipid metabolism and ER stress as targetable vulnerabilities in renal cell carcinoma

**DOI:** 10.1371/journal.pone.0172632

**Published:** 2017-03-15

**Authors:** Ayse Batova, Diego Altomare, Kim E. Creek, Robert K. Naviaux, Lin Wang, Kefeng Li, Erica Green, Richard Williams, Jane C. Naviaux, Mitchell Diccianni, Alice L. Yu

**Affiliations:** 1 Department of Pediatrics, University of California, San Diego, California, United States of America; 2 Department of Drug Discovery and Biomedical Sciences, South Carolina College of Pharmacy, University of South Carolina, Columbia, South Carolina, United States of America; 3 The Mitochondrial and Metabolic Disease Center, Department of Pathology, University of California, San Diego, San Diego, California, United States of America; 4 Department of Medicine, University of California, San Diego, California, United States of America; 5 Institute of Stem Cell and Translational Cancer Research, Chang Gung Memorial Hospital, Linkou, Taoyuan, Taiwan; Duke University School of Medicine, UNITED STATES

## Abstract

Renal cell carcinoma (RCC) is among the top ten most common forms of cancer and is the most common malignancy of the kidney. Clear cell renal carcinoma (cc-RCC), the most common type of RCC, is one of the most refractory cancers with an incidence that is on the rise. Screening of plant extracts in search of new anti-cancer agents resulted in the discovery of englerin A, a guaiane sesquiterpene with potent cytotoxicity against renal cancer cells and a small subset of other cancer cells. Though a few cellular targets have been identified for englerin A, it is still not clear what mechanisms account for the cytotoxicity of englerin A in RCC, which occurs at concentrations well below those used to engage the targets previously identified. Unlike any prior study, the current study used a systems biology approach to explore the mechanism(s) of action of englerin A. Metabolomics analyses indicated that englerin A profoundly altered lipid metabolism by 24 h in cc-RCC cell lines and generated significant levels of ceramides that were highly toxic to these cells. Microarray analyses determined that englerin A induced ER stress signaling and an acute inflammatory response, which was confirmed by quantitative PCR and Western Blot analyses. Additionally, fluorescence confocal microscopy revealed that englerin A at 25 nM disrupted the morphology of the ER confirming the deleterious effect of englerin A on the ER. Collectively, our findings suggest that cc-RCC is highly sensitive to disruptions in lipid metabolism and ER stress and that these vulnerabilities can be targeted for the treatment of cc-RCC and possibly other lipid storing cancers. Furthermore, our results suggest that ceramides may be a mediator of some of the actions of englerin A. Lastly, the acute inflammatory response induced by englerin A may mediate anti-tumor immunity.

## Introduction

Renal cell carcinoma (RCC) is among the top ten most common forms of cancer and is the most common malignancy of the kidney [1]. Overall, the lifetime risk for developing kidney cancer is about 1 in 63 (1.6%) according to the American Cancer Society. Clear cell renal cell carcinoma (cc-RCC) is the most common type of RCC with an incidence that is on the rise for reasons that are not entirely clear [1]. The treatment options for RCC are surgery, radiation therapy (palliative), targeted therapy (bevacizumab, sunitinib, sorafenib, everolimus, temsirolimus), biological therapy (immunotherapy), and combinations of these [2,3]. Though surgical resection can be curative in patients who suffer from localized RCC, diagnosis is frequently made when the disease has progressed and cannot be resected. Furthermore, cc-RCC is one of the most radio- and chemo-resistant cancers and no curative treatment is available for metastatic cc-RCC [4]. As of 2011, the two-year survival rate for patients with metastatic disease was reported to be under 20% despite approved targeted therapies [5]. However, the very recent approval of two new multi-targeted agents is anticipated to improve this survival rate as these new agents have yielded superior median overall survival in patients with metastatic RCC compared to the previously approved single target agent, everolimus, in recent clinical trials [6,7]. However, toxicities were associated with these new agents. Hence, there is an urgent need to investigate novel agents that take advantage of the unique biology of RCC.

The fact that mutation in each of the RCC susceptibility genes results in the dysregulation of at least one metabolic pathway suggests that RCC is a metabolic disease [8–10]. There is increasing evidence supporting this notion including a recent study that found that the majority of proteins dysregulated in cc-RCC, were proteins involved in glucose and lipid metabolism [11]. Moreover, a recent landmark study conducted metabolic profiling of patient samples with matched normal tissue and found a network of metabolic shifts associated with the genesis and progression of cc-RCC tumors [12]. Though many tumors rely on increased glucose uptake and glycolysis, few accumulate lipids to the extent of cc-RCC giving it its distinct clear cell phenotype. The presence of extensive lipid droplets in cc-RCC suggests that cc-RCC has a profoundly altered lipid metabolism compared to normal cells. This notion is supported by increasing evidence including the finding of aberrant expression of fatty acid synthase, steroyl-CoA desaturase 1 (SCD1), A:cholesterol acyl transferase, glucosylceramide synthase and several other lipogenic genes in cc-RCC [13–15]. These findings imply that novel agents which take advantage of this unique biology may be effective in the treatment of this chemo- and radio-resistant cancer.

Natural products possess enormous structural and chemical diversity and are a rich source of drugs or drug-like leads. Although the final products may not necessarily represent the active ingredients of the natural source, the majority of all drugs in the market have their origin in nature [16,17]. Englerin A is guaiane sesquiterpene that was isolated from the Tanzanian plant *Phyllanthus engleri* and has attracted much attention because of its unique structure and cytotoxicity profile in cancer cells. Englerin A was documented to have potent cytotoxicity preferentially against renal cancer cells [18–20] when tested against the NCI60 cell panel. Very recently, however, a study which screened englerin A against 524 cancer cell lines from the cancer cell line encyclopedia reported that englerin A was also cytotoxic to a small subset of cancer cells in addition to renal cancer cells [21]. Importantly, englerin A had no toxicity towards normal cells even at concentrations greater than 1 x 10^6^-fold greater than the IC50 in cancer cells sensitive to englerin A [19]. In addition to its unique structure and cytotoxicity profile, englerin A is distinctive because it can cause cell death by multiple death mechanisms including necrosis and apoptosis, with apoptosis occurring in the absence of caspase activation [19,22]. The distinctive features of englerin A suggest that it has a unique mechanism of action and recent reports, though lacking consensus, support this notion. Englerin A has been shown to activate protein kinase C theta (PKCθ) and was proposed to affect cell viability by promoting glucose dependence while simultaneously starving cells of glucose [23]. Englerin A has also been shown to increase cytosolic calcium levels which may play a role in the decreased phosphorylation and activity of the oncoprotein, EWS-FLI1, in Ewing’s sarcoma [24]. Two other groups have reported that englerin A inhibits tumor cell growth by activating the transient receptor potential cation channel, subfamily C, member 4 (TRPC4) ion channel, while a third group reported that englerin A antagonized L-type calcium channels [21,25,26]. However, in at least two of these studies, the concentration of englerin A that was required to modulate these calcium channels was much higher than that required to kill renal carcinoma cells and other cells sensitive to the cytotoxic effects of englerin A. For instance, the most recent study reported that englerin A displaced a radiolabeled tool compound bound to an L-type calcium channel in a concentration-dependent manner with a Ki of 5.7 μM. In contrast, the EC_50_ of englerin A in reducing the viability or growth of most renal cancer cell lines in the NCI60 cell panel is less than 60 nM, suggesting that modulation of L-type or TRPC calcium channels is not required for englerin A to induce toxic effects in these cells [18–20,27]. It is still not clear what mechanisms account for the cytotoxicity of englerin A at nanomolar levels in these cells. We anticipate that there may be other mechanisms and targets of englerin A as natural products generally have multiple targets. In the current study, we chose a systems biology approach to explore the mechanism(s) of action of englerin A at a more global level. Our findings indicate that englerin A profoundly alters lipid metabolism generating ceramides, ER stress, and a strong inflammatory response. Furthermore, our results suggest that ceramides may be a mediator of some of the actions of englerin A.

## Materials and methods

### Cell culture

A498 cells were purchased from ATCC in 2012 and UO-31 cells were obtained from the NCI in 2016. Cell lines were authenticated by morphology and by their sensitivity to englerin A cytotoxicity. Both cell lines were maintained in RPMI medium supplemented with 10% FBS, 2 mM L-glutamine, and 100 units/ml penicillin/streptomycin (complete RPMI). All experiments were conducted with cells of passage number less than 25.

### Reagents

Englerin A was purchased from Ceriliant Corporation and Cfm Oskar Tropitzsch. C8- and C16-ceramides and C8-ceramide-1-phosphate were obtained from Avanti Polar Lipids. Imipramine hydrochloride and Cambinol were purchased from Sigma and Cayman Chemical, respectively.

### Metabolomics analysis

#### Cell treatment and extraction

A498 cells were plated in 100mm dishes at 0.5 x 10^6^ and 1 x 10^6^ cells/dish in complete RPMI for control and treated cells, respectively. The following day, cells were refed with complete RPMI containing 0.1% DMSO or 100 nM englerin A in DMSO with each condition being conducted in quadruplicate. Cells were incubated with vehicle or englerin A for 24 or 48 h and then they were snap frozen with liquid nitrogen. Cell extracts were obtained by the addition of 1 ml of ice-cold methanol:water (80:20) to each dish followed by scraping cells into 1.7ml Eppendorf tubes and vortexing vigorously for 30 sec. A 50 ul aliquot of sample was removed for the picogreen DNA assay for purposes of biomass normalization, and the remaining sample was kept at -80°C until ready for metabolic analysis.

#### Metabolomics

Samples were analyzed on an AB SCIEX QTRAP 5500 triple quadrupole mass spectrometer equipped with a Turbo V electrospray ionization (ESI) source, Shimadzu LC-20A UHPLC system, and a PAL CTC autosampler. Typically, 300 μl of cells in methanol:water (80:20) was thawed on ice and transferred to a 1.7 ml Eppendorf tube. Five μl of a cocktail containing 25–35 commercial stable isotope internal standards, and 5.0 μl of 57 stable isotope internal standards that were custom-synthesized in E. coli and S. cerevisiae by metabolic labeling with ^13^C-glucose, and ^13^C-bicarbonate, were added, and vortexed vigorously for 30 sec. Macromolecules (protein, DNA, RNA, glycans, etc.) then were removed by centrifugation at 16,000g x 10 min at 4°C. The supernatants containing the extracted metabolites and internal standards were transferred to labeled cryotubes and stored at -80°C for LC-MS/MS analysis. LC-MS/MS analysis was performed by scheduled multiple reaction monitoring (sMRM) under Analyst v1.6.2 software control in both negative and positive mode with rapid polarity switching (50 ms). Nitrogen was used for curtain gas (set to 30), collision gas (set to high), ion source gas 1 and 2 (set to 35). The source temperature was 500°C. Spray voltage was set to -4500 V in negative mode and 5500 V in positive mode. The values for Q1 and Q3 mass-to-charge ratios (m/z), declustering potential (DP), entrance potential (EP), collision energy (CE), and collision cell exit potential (CXP) were determined and optimized for each MRM for each metabolite. Ten microliters of extract was injected by PAL CTC autosampler via a 10 μl stainless steel loop into a 250 mm × 2.0 mm, 4 μm polymer based NH2 HPLC column (Asahipak NH2P-40 2E, Showa Denko America, Inc., NY) held at 25°C for chromatographic separation. The mobile phase was solvent A: 95% water with 20 mM (NH_4_)_2_CO_3_ (Sigma, Fluka Cat# 74415-250G-F), 5% acetonitrile, and 38 mM NH_4_OH (Sigma, Fluka Cat# 17837-100ML), final pH 9.75; solvent B: 100% acetonitrile. Separation was achieved using the following gradient: 0–3.5 min: 95% B, 3.6–8 min: 85% B, 8.1–13 min: 75% B, 13.5–35 min: 0% B, 36–46 min: 95% B, 46.1 min: end. The flow rate was 200 μl/min. Pump pressures ranged from 920–2600 psi over the course of the gradient. All the samples were kept at 4°C during analysis. The chromatographic peaks were identified using MultiQuant (v3, AB Sciex), confirmed by manual inspection, and the peak areas integrated. Over 363 metabolites were detectable in all samples.

#### Data analysis

Metabolomic data were normalized by DNA concentration measured with the picogreen assay then log-transformed, scaled by control standard deviations, and analyzed by multivariate partial least squares discriminant analysis (PLSDA), principal components analysis (PCA), t test, univariate ANOVA with pairwise comparisons and post hoc correction for multiple hypothesis testing using Fisher’s least significant difference method in MetaboAnalyst [28], or the false discovery rate (FDR) method of Benjamini and Hochberg [29]. Metabolites with variable importance in projection (VIP) scores determined by PLSDA that were greater than 1.5 were considered significant. Significant metabolites were grouped into pathways and their VIP scores summed to determine the rank-ordered significance of each biochemical pathway.

### Microarray analysis

#### Cell culture and RNA extraction

A498 cells were plated in complete RPMI onto 100 mm dishes at 0.9 x 10^6^ cells per dish. After cells were allowed to adhere overnight, cells were refed with complete RPMI containing 0.1% DMSO (vehicle) or 100 nM englerin A. Cells were then incubated with englerin A or DMSO for 3, 8, and 20 h prior to isolation of RNA. Cell extracts were prepared using 1 ml of TRI Reagent (Zymo Research, Irvine, CA) per dish. Total RNA was then purified from cell extracts using spin columns by Zymo Research according to the manufacturer’s directions.

#### mRNA labeling and hybridization

Microarrays experiments were performed using the Agilent platform. Total RNA was amplified and labeled using Agilent’s Low Input Quick Amp Labeling Kit (Cat. # 5190–2306) according to the manufacturer’s recommendations. Briefly, mRNA contained in 200 ng of total RNA was converted into cDNA using a poly-dT primer that also contained the T7 RNA polymerase promoter sequence. Subsequently, T7 RNA polymerase was added to cDNA samples to amplify original mRNA molecules and to simultaneously incorporate cyanine-3 labeled CTP (cRNA) into the amplification product. In the next step, labeled RNA molecules were purified using Qiagen’s RNeasy Mini Kit (Cat. # 74104). After spectrophotometric assessment of dye incorporation and cRNA yield, samples were stored at -80°C until hybridization. Labeled cRNA samples (600 ng) were hybridized to SurePrint G3 Human Gene Expression 8x60K v2 Microarrays (Cat. # G4858A-039494) at 65°C for 17 h using Agilent’s Gene Expression Hybridization Kit (Cat. # 5188–5242) according to the manufacturer’s recommendations. After washes, arrays were scanned using a High Resolution Agilent DNA Microarray Scanner System (Cat. # G2565CA) and the images saved in TIFF format.

#### Data analysis

Data was extracted from images with Feature Extractor Software version 10.7.3.1 (Agilent) where background correction was also performed. Subsequently, background-corrected data was uploaded into GeneSpring GX version 11.5.1 for analysis. In this process, data was log2 transformed, quantile normalized and base line transformed using the median of all samples. Then, the data was filtered by flags in a way that 75% of the samples in at least one of the treatment groups have a “detected” flag. Differentially expressed genes were determined by analysis of the data using a moderated T test. Cutoff values of 0.01 and 1.5 were used for p-value and fold change, respectively. GeneSpring and Ingenuity pathway analysis software were used to perform pathway analysis and biological contextualization of the differentially expressed genes. The pathway determination using Ingenuity software was conducted by analysis of differentially expressed genes for all three time points (3, 8, 20 h), analyzed together.

### Reverse transcription and quantitative real time PCR

Culture of A498 cells and RNA extraction were carried out as described above for microaray analysis. In independent experiments, UO-31 cells were treated with 0.1% DMSO or 40 nM englerin A for 3 h and 8 h followed by cell harvesting and extraction of RNA as described above.

Reverse transcription was carried out using the iScript cDNA Synthesis Kit (BioRad, Hercules, CA). Each reaction contained a final volume of 20 μL including 4 μL iScript RT Supermix, 1 μg RNA, and nuclease-free water. All reverse transcription assays were conducted on the Applied Biosystems GeneAmp PCR System 9700 (ThermoFisher Scientific, Waltham, MA). The conditions for the reverse transcription assay were as follows: priming for 5 min at 25°C, reverse transcription for 30 min at 42°C, and inactivation of reverse transcriptase for 5 min at 85°C. Real time PCR was performed using iQ SYBR Green Supermix (BioRad). The cycling conditions for the real time PCR for all genes except EIF4A2, were as follows: initial denaturation of template at 95°C for 3 min, followed by 45 cycles of denaturation at 95°C for 10 sec, and primer annealing/extension at 58°C for 30 sec. The annealing temperature for the EIF4A2A reaction was 56.2°C while all other conditions were the same as with other reactions. A melt curve was also generated for each gene to confirm primer specificity and to ensure generation of only one product. All products generated were melted at 95°C then annealed at 55°C and subjected to gradual increases in temperature. The specific cycling conditions were as follows: denaturation at 95°C for 1 min, annealing at 55°C for 1 min, and repeated 81 cycles for 10 sec at 55°C. The DNA sequences of the primers used for real time PCR were as follows: ATF4: forward, 5’-CCC CCT TCA CCT TCT TAC AA-3’, reverse, 5’-AAG GGG TGT CTT CCT CCT TT-3’; CXCL2: forward, 5’-CAC TCA AGA ATG GGC AGA AA-3’, reverse, 5’-CCT CTG CAG CTG TGT CTC TC-3’; EGR1: forward, 5’-TGA CCG CAG AGT CTT TTC CT-3’, reverse, 5’-TGG GTT GGT CAT GCT CAC TA-3’; EIF4A2: forward, 5’-CCG GGA GAG TGT TTG ATA TG-3’, reverse, 5’-ACA CAT CAG TTG GCA TTG TG-3’; IL6: forward, 5’-ATG CAA TAA CCA CCC CTG AC-3’, reverse, 5’-GAG GTG CCC ATG CTA CAT TT-3’; PPP1R15A: 5’-CAG GTC CTG GGA GTA TCG TT-3’, reverse, 5’-GGG AGG ACA CTC AGC TTC TC-3’; PPP1527: forward, 5’-GGT ACC TGG GGT GTA AAG GTC-3’, reverse, 5’-TAC ACA GGC TTG AAC CCG AC-3’; TNFα: forward, 5’-TCC TTC AGA CAC CCT CAA CC-3’, reverse, 5’-AGG CCC CAG TTT GAA TTC TT-3’. GAPDH (internal control): forward, 5’-GAA GGT GAA GGT CGG AGT-3’, reverse, 5’-GAA GAT GGT GAT GGG ATT TC-3’.

### Western blot analysis

A498 cells were plated in complete RPMI at 1.2 x 10^6^ and 2.0 x 10^6^ cells per T-75 flask for control and treated cells, respectively. After allowing cells to attach overnight, cells were treated with 0.1% DMSO or 100 nM englerin A for 3, 8, and 24 h. Cell extracts were prepared and Western Blot analysis was conducted as described previously [22]. Antibodies against MKK4, p-MKK4, eIF2α, p- eIF2α, p-JNK, and B-actin were obtained from Cell Signaling Technology (Danvers, MA) and that against JNK was obtained from Abcam (Cambridge, MA). Except for anti-JNK (diluted 1:2000), all antibodies were diluted 1:1000. Bands were visualized using SignalFire Plus ECL enhanced chemiluminescent substrate (Pierce) and exposed to HyBlot CL film (Denville Scientific). The film was developed with a Kodak film developer.

### [^3^H]-thymidine incorporation assay

A498 or UO-31 cells were plated in complete RPMI at 3,500 or 2,000 cells per well of a 96-well plate, respectively. After allowing cells to attach overnight, cells were treated with 0.1% DMSO (vehicle), or increasing concentrations of englerin A, C8-ceramide, or C8-ceramide-1-phosphate and incubated for 48 h. All conditions were conducted in triplicate. Cells were pulsed with [^3^H]-thymidine (1.6 μCi/well) during the last 7 h of incubation and then trypsinized and deposited onto glass microfiber filters using the Brandel M 24 cell harvester (Gaithersburg, MD). Incorporation of [^3^H]-thymidine into DNA was then determined by counting in a scintillation counter (Beckman Coulter, Fullerton, CA).

### Fluorescent confocal microscopy

UO-31 cells were plated in complete RPMI onto coverslips (4.9 cm^2^) placed in 6-well plates at 20,000 and 40,000 cells per cover slip for control and treated cells, respectively. In separate experiments, A498 cells were plated at 65,000 cells per 2-well chamber slides. After cells were allowed to attach overnight, cells were refed with complete RPMI containing 0.1% DMSO (vehicle) or with englerin A at 25, 50 or 100 nM. Cells were incubated for 28.5 h with englerin A or vehicle and then rinsed once with assay buffer (Enzo Life Sciences, Farmingdale, NY), and fixed with 3.7% formaldehyde in assay buffer for 15 min at 37°C. Formaldehyde solution was prepared from a 16% formaldehyde stock (Thermo Scientific, Rockford, IL). Coverslips were then washed 3 times with assay buffer before staining with ER-ID^®^ Green and Hoechst stain (0.8 μl of each stain per ml of assay buffer) for 25 min using the ER-ID^®^ Green assay kit from Enzo Life Sciences (Farmingdale, NY). After staining, cover slips were washed 3 times with assay buffer followed by two rinses in distilled water and then were mounted onto slides using ProLong Diamond mounting medium (Thermo Fisher Scientific, Waltham, MA). High resolution images were acquired with a Nikon A1R confocal microscope using a 488 nm excitation laser and a 100x oil objective. Images were captured at Nyquist resolution in the XY plane and the pinhole was adjusted to image at an optical depth of field of 0.3 microns in the Z axis.

## Results

### Effects of englerin A on metabolism

**Fig 1:**
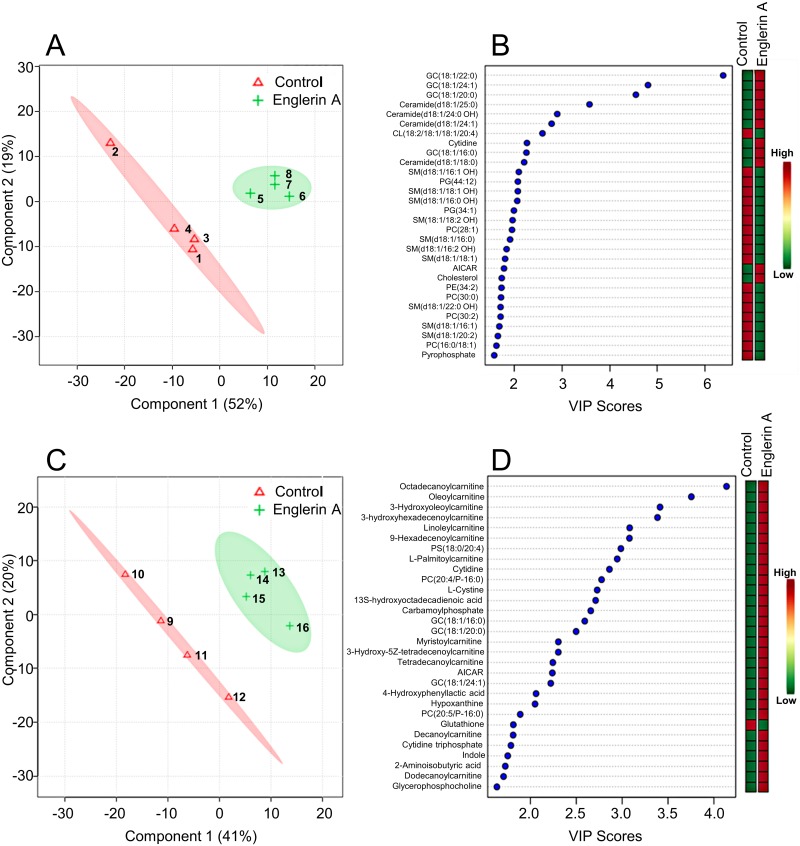
Multivariate analysis of metabolites altered by englerin A in renal cancer cells. Multivariate analysis using partial least squares discriminant analysis (PLSDA) clearly distinguished control and englerin A treated groups of A498 cells at both 24 h (A and B) and 48 h (C and D) durations of treatment. Panels B and D list the top metabolites and their VIP scores and indicate how they are altered compared to control. Numbers within shaded areas in graphs A and C represent samples within control and treated groups.

Englerin A, although highly toxic to most renal cancer cell lines in the NCI60 cell panel, requires greater than 24 h to induce significant cell death at concentrations greater than the EC_50_ [22] in viability assays. This is in contrast to many chemotherapeutic agents such as camptothecin and doxorubicin that can induce significant levels of apoptosis in less than 8 h [30,31]. The kinetics of englerin A induced cell death indicates that multiple choreographed events are required for cell death to take place. In line with this, we have previously demonstrated that englerin A induces authophagy as a survival mechanism that ultimately fails and results in cell death [22]. Taken together, our previous results suggested that englerin A induced multiple orchestrated events leading to metabolic stress and cell death. To further explore and study these events leading to metabolic stress and cell death, we conducted metabolomics analyses given that metabolomics is the collective cellular chemistry representing the functional interaction of genes and the environment. For these experiments, A498 cells, one of the most sensitive renal carcinoma cell lines to englerin A cytotoxicity, were treated with vehicle (0.1% DMSO) or 100 nM englerin A for 24 and 48 h. The metabolomics data set has been deposited into the Metabolomics Workbench repository (http://dev.metabolomicsworkbench.org:22222/data/DRCCMetadata.php?Mode=Study&StudyID=ST000465). Multivariate analysis was used to identify the chemical differences between englerin A treated and control cells. As shown in Fig 1A and 1C, these studies found that the metabolic profile of englerin A treated cells was very distinct from vehicle treated control cells. The biochemical pathways and metabolites that were altered by englerin A in A498 cells were ranked and tabulated in Table 1 and Fig 1B and 1D. For 24 h of englerin A treatment, the main finding from pathway analysis was that changes in sphingolipids contributed to 43% of all the disturbances in metabolism associated with englerin A (Table 1). Furthermore, changes in phospholipids contributed another 23% and changes in gangliosides contributed another 22% to the metabolic disturbances associated with englerin A treatment (Table 1). This indicates that the largest metabolic disturbance (88%) associated with englerin A at 24 h is due to changes in lipid metabolism. In particular, among the top metabolites, several ceramides (4 species) and glucosylceramides (4 species) increased up to 18- and 178-fold, respectively (Fig 1B and S1 Table). On the other hand, all sphingomyelins (10 species) decreased up to 5.5-fold. The increase in ceramides upon treatment with englerin A is a clear sign of cell stress as ceramides are generated in response to cell stress and mediate autophagy and cell death [32,33]. Ceramides are synthesized acutely from sphingomyelins through the action of stress-induced acid and neutral sphingomyelinases. Ceramides are also known to inhibit the respiratory complex III, thereby inhibiting all aerobic energy production from glucose and fatty acids [34], and to form channels in the mitochondrial membrane, activating the intrinsic arm of programmed cell death [35]. Furthermore, ceramides are also known to promote ER stress [36,37]. In addition to the increase in ceramides, several phospholipids including phosphatidylcholine, phosphatidylglycerol, and phosphatidylethanolamine phospholipids were decreased about 3- to 5-fold in response to englerin A by 24 h (Fig 1B and S1 Table). The depletion in these phospholipids may also result in ER stress and disrupted Golgi trafficking as previously observed with the depletion of phosphatidylcholine [38]

**Table 1:** Top metabolic pathways impacted by englerin A in renal cancer cells.

**Pathway Name (24 h)**	**Measured Metabolites in the Pathway (N)**	**Expected Pathway Proportion (P = N/599)**	Expected Hits in Sample of 37 (P * 37)	**Observed Hits in the Top 37 Metabolites**	**Fold Enrichment (Obs/Exp)**	**Impact (Sum VIP Score)**	**Fraction of Impact (VIP Score) Explained (% of 81.7)**	**Increased**	**Decreased**
Sphingolipid Metabolism	74	0.123	4.6	17	3.7	34.9	43%	4	13
Phospholipid Metabolism	110	0.184	6.8	11	1.6	18.9	23%	0	11
Ganglioside Metabolism	12	0.020	0.7	4	5.4	18.0	22%	4	0
Cardiolipin Metabolism	12	0.020	0.7	1	1.3	2.6	3%	0	1
Pyrimidine Metabolism	26	0.043	1.6	1	0.6	2.3	3%	1	0
Purine Metabolism	34	0.057	2.1	1	0.5	1.8	2%	1	0
Cholesterol, Cortisol, Non-Gonadal Steroid Metabolism	21	0.035	1.3	1	0.8	1.7	2%	1	0
Phosphate and Pyrophosphate Metabolism	1	0.002	0.1	1	16.2	1.6	2%	0	1
**Pathway Name (48 h)**	**Measured Metabolites in the Pathway (N)**	**Expected Pathway Proportion (P = N/599)**	Expected Hits in Sample of 34 (P * 34)	**Observed Hits in the Top 34 Metabolites**	**Fold Enrichment (Obs/Exp)**	**Impact (Sum VIP Score)**	**Fraction of Impact (VIP Score) Explained (% of 82.7)**	**Increased**	**Decreased**
Fatty Acid Oxidation and Synthesis	47	0.078	2.7	14	5.2	38.7	47%	14	0
Phospholipid Metabolism	110	0.184	6.2	5	0.8	10.8	13%	5	0
Pyrimidine Metabolism	26	0.043	1.5	5	3.4	10.5	13%	5	0
Ganglioside Metabolism	12	0.020	0.7	3	4.4	7.3	9%	3	0
SAM, SAH, Methionine, Cysteine, Glutathione Metabolism	21	0.035	1.2	2	1.7	4.5	5%	1	1
Purine Metabolism	34	0.057	1.9	2	1.0	4.3	5%	2	0
Eicosanoid and Resolvin Metabolism	22	0.037	1.2	1	0.8	2.7	3%	1	0

Pathways impacted by Englerin A were determined using MetaboAnalyst 3.0 software after measuring metabolites from A498 cells treated with 0.1% DMSO or englerin A at 100 nM for 24 h and 48 h.

For 48 h of englerin A treatment, the main findings from pathway analysis was that an increase (up to 15.5-fold) in the levels of fatty acylcarnitine, metabolites associated with lipid oxidation, contributed to 47% of the metabolic perturbations associated with englerin A (Table 1 and S2 Table). The reason for the increase in carnitine derivatives is likely due to mitochondrial dysfunction and the inability to import acyl carnitines into mitochondria for beta oxidation and ATP synthesis by mitochondrial oxidative phosphorylation. Interestingly, a phosphatidylserine phospholipid was elevated about 7-fold (S2 Table) in response to englerin A. Under healthy growth conditions, mitochondria receive phosphatidylserine lipids from the ER at metabolic synapses, structured contact points in the ER called mitochondria associated membranes. Phosphatidylserine lipids are then decarboxylated in the mitochondria to produce phosphatidylethanolamine lipids destined for multiple metabolic fates. When mitochondria are dysfunctional, phosphatidylserine phospholipids accumulate. Purine and pyrimidine precursors of RNA and DNA synthesis were increased at 48 h by englerin A contributing to 5% and 13% of metabolic changes associated with englerin A, respectively (Table 1 and Fig 1D). Specifically, among the top metabolites, AICAR, hypoxanthine, cytidine, cytidine triphosphate and thymidine were increased suggesting a state of low energy and the inability to incorporate purine and pyrimidine monomers into polymers of RNA and DNA (Fig 1D). Finally, among the top metabolites, the one metabolite that was decreased was reduced glutathione (3.3-fold), indicative of oxidative stress (Fig 1D and S2 Table). This finding is in agreement with that of a previous report showing englerin A induced production of reactive oxygen species renal cancer cells [19]. Hence, 48 h of englerin A treatment resulted in mitochondrial dysfunction and oxidative stress as well the inability to synthesize RNA and DNA. Overall, our results indicate a profound alteration of lipid metabolism with the generation of ceramides as an initial response to cell stress resulting in mitochondrial dysfunction and, likely, ER stress, ultimately leading to cell death. Furthermore, our results highlight ceramides as potential key mediators of englerin A induced cell stress and cell death.

### Englerin A induces ER stress and an unfolded protein and inflammatory response

The key finding of the generation of ceramides by englerin A would suggest specific stress signaling associated with ceramides. For instance, ceramides are known to induce ER stress resulting in the unfolded protein response as well as to participate in autophagic response signaling [39]. Hence, to extend and further confirm our findings from metabolomic analyses we conducted microarray analysis using the Agilent platform (SurePrint G3 Human Gene Expression 8x60K v2 Microarrays). For this study, A498 cells were treated with 100 nM englerin A or vehicle (0.1% DMSO) for 3, 8, and 20 h to examine early, intermediate, and late events. The microarray data set has been deposited into the GEO repository (http://www.ncbi.nlm.nih.gov/geo/query/acc.cgi?acc=GSE86047). The list of differentially expressed genes at the three different durations of englerin A treatment relative to control are presented in S3 Table. Both GeneSpring and Ingenuity software were used for pathway analysis. The top most significant pathways modulated by englerin A are listed in Table 2. The pathways that are labeled with an asterisk (*) in Table 2 are those that were determined by both GeneSpring and Ingenuity software. For the most part, the pathways determined by each of the software were either related pathways or the same pathways. The top pathway modulated by englerin A was ErbB1 downstream signaling. The ErbB receptors signal through Akt, MAPK, and other pathways to regulate cell proliferation, migration, differentiation, apoptosis, and cell motility. Previously, we identified that englerin A inhibited the phosphorylation of both Akt and Erk [22], in agreement with modulation of downstream ErbB1 signaling. Both pathway analysis software identified the AP1 transcription factor network to be modulated by englerin A at 3h. This network also includes the ATF2 transcription factor which is upregulated by englerin A at 3 h. Members of this transcription factor network are involved in cell stress signaling and are activated by stress kinases such as JNK and p38 [40,41]. This stress signaling is likely due to ER stress as PERK regulated gene expression is induced by englerin A within 3 h. PERK is a type I membrane protein located in the endoplasmic reticulum, where it is induced by ER stress caused by misfolded proteins [42]. Ceramides are an established ER stressor that can lead to an unfolded protein response [39,43]. Though not among the top pathways regulated by englerin A at the gene level, pathway analysis by GeneSpring did identify ceramide signaling among pathways modulated by englerin A (P = 0.0057, 5/45 genes regulated). This finding is in agreement with our metabolomics data suggesting ceramides as a mediator of englerin A action. Several additional findings support this notion. For instance, englerin A upregulated TNF expression by 15-fold (S3 Table) thereby regulating TNF signaling, a pathway also known to be modulated by ceramides [44]. Ceramides are reported to result in cell death via interaction with members of the TNF superfamily of receptors[44,45]. Ceramide promoted ER stress is known to activate Toll like receptor (TLR) signaling and inflammation [46]. In agreement with this, pathway analysis by both GeneSpring and Ingenuity determined that englerin A induced TLR signaling by 3 h (Table 2). Collectively, these results suggest that ceramides generated in response to englerin A induce ER stress resulting in an unfolded protein response and inflammatory signaling by TNF and TLR.

**Table 2:** Top cell signaling pathways regulated by englerin A in renal cancer cells.

Pathway	P-Value	Number of Genes Regulated in Pathway	Number of Pathway Genes in Experiment
**3 h of EA treatment**			
ERbB1 downstream signaling	6.76 x 10^−5^	15	168
AP-1 transcription factor network*	1.31 x 10^−4^	5	20
TLR signaling*	1.35 x 10^−4^	7	42
ATF2 transcription factor network	1.39 x 10^−4^	8	57
PDGFR-B signaling	2.71 x 10^−4^	13	150
TGF-ßR signaling	2.97 x 10^−4^	12	132
Golgi associated vesicle biogenesis	3.32 x 10^−4^	4	13
Nectin adhesion	4.76 x 10^−4^	13	159
TNF signaling*	6.66 x 10^−4^	3	7
PERK regulated gene expression	6.66 x 10^−4^	3	7
**8 h of EA treatment**			
ERbB1 downstream signaling	4.00 x 10–5	19	168
ATF2 transcription factor network	6.13 x 10^−5^	10	57
PDGFR-B signaling	9.72 x 10^−5^	17	150
IL-12 mediated signaling	1.13 x 10^−4^	9	49
Amb2 integrin signaling	1.2 x 10^−4^	6	21
Nectin adhesion	1.98 x 10^−4^	17	159
Nuclear ERß network	2.61 x 10^−^4	4	9
**20 h of EA treatment**			
RIG-I/MDA5 mediated* induction of IFN-alpha/beta pathways	3.46 x 10^−6^	6	31
TLR signaling	2.17 x 10^−5^	6	42
IL-12 signaling	5.34 X 10^−5^	6	49
Cytokine Signaling*	7.09 x 10^−5^	4	18
ATF2 transcription factor network	1.14 x 10^−4^	6	57
IL-23 signaling	1.99 x 10^−4^	3	9
Cytokine-cytokine receptor interaction*	2.38 x 10^−4^	10	185
RIG1-like receptor signaling	4.04 x 10–4	4	26

Microarrays experiments were performed using Agilent’s platform. Probes that achieved a significance of P = 0.01 or less in the ANOVA analysis with Benjamin-Hochberg correction were subjected to pathway analysis by GeneSpring and Ingenuity Pathway Analysis software. A fold change of 1.5 or greater was used in these analyses. For pathway analysis using Ingenuity software, the differentially expressed genes for all three time points (3, 8, 20 h) were analyzed simultaneously.

* Pathways labeled with an asterisk (*) are those determined by both Ingenuity and GeneSpring software.

Because of their association with Jun, Fos, ATF3 and other members, additional pathways that may be modulated by engerin A at 3 h also included the PDGF and TGF-ß signaling pathways (Table 2). Importantly, both of these pathways signal through ERK1/2 which is inhibited by engerin A [22]. Lastly, englerin A was determined to modulate nectin adhesion as well as Golgi-associated vesicle biogenesis, the later which is required for cytokine secretion and is consistent with TLR signaling.

As shown in Table 2, several of the pathways regulated by englerin A at 8 h include those also modulated at 3 h. Additional pathways potentially affected by englerin A at 8 h include IL-12 and Amb2 integrin signaling, as well as signaling associated with the nuclear ERß network. Englerin A induces the expression of several interleukin genes and their receptors thus affecting their associated signaling including that of IL-12 (S3 Table). Members of the amb2 integrin pathway include NFκB, AKT, TNF and IL-6, all of which are modulated by englerin A at the gene or protein level [22] (S3 Table). Lastly, The ERß receptor is known to interact with other transcription factors such as AP1 through transcription factor cross talk. Hence modulating the expression of members of the AP1 network by englerin A may regulate ERß signaling [47].

Pathway analysis indicated that at 20 h, englerin A clearly induced an inflammatory response akin to the response to pathogens through pattern recognition receptors belonging to the RIG-1 like family (Table 2). These responses included signaling by interferon α/ß as well as signaling by inflammatory interleukins including IL-23 and IL-12. Furthermore, the expression of several inflammatory chemokines including CCL4, CXCL2, and CCL20 were also induced by englerin A by up to 34-fold (S3 Table). Although the expression of several interleukin genes were elevated prior to 20 h, interferon signaling only became apparent at 20 h suggesting a gradual build to an inflammatory response culminating in interferon signaling. In summary, gene expression profiling and pathway analysis have revealed that englerin A induced ER stress signaling, also known as the unfolded protein response, likely due to the generation of ceramides as well as to the depletion of certain phospholipids. This response involved both PERK and the AP1 transcription factor network, the latter, which is activated by the stress kinase, JNK. Secondly, englerin A induced an inflammatory response involving signaling by TNF, interleukins, as well as that by interferon α/ß. These findings are in agreement with different lines of research revealing that pathways activated by ER stress such as the unfolded protein response induce sterile inflammation in various cells [46,48].

In order to confirm our findings from microarray analysis regarding englerin A induced ER stress signaling and inflammation we conducted quantitative PCR analyses using both A498 and UO-31 renal carcinoma cell lines. In these experiments, A498 and UO-31 cells were treated for 3–20 h with 100 nM and 40 nM englerin A, respectively. Control cells were treated with 0.1% DMSO. The mRNA levels of several genes associated with ER stress-induced unfolded protein response signaling including ATF4, EGR1, EIF4A2, and PPP1R1A were analyzed. EIF4A2 which is involved in EIF2 and eIF4/p70S6K signaling in the initiation of translation [49] was upregulated over 2-fold in both A498 and UO-31 cells, in agreement with microarray results (Table 3). Activating transcription factor 4 (ATF4) is a component of the PERK arm of ER stress signaling that drives the expression of CHOP and GADD34, also known as PPP1R15A. The expression of both ATF4 (up to 3.3-fold) and GADD34/PPP1R15A (up to 11.3-fold) were upregulated by englerin A in both cell lines at 3 h and at 8 h. Furthermore, the expression of EGR1 which encodes a transcription factor regulated by PERK and plays a crucial role in stress induced cytokine production [50] was increased in both A498 and UO-31 cells by up to about 32-fold, with the greatest increase occurring at 3 h consistent with microarray results. Real time RT-PCR analysis of inflammatory cytokines and chemokines including TNFα, IL-6, and CXCL2 revealed dramatic increases (up to 56.7-fold) in their expression in response to englerin A in both A498 and UO-31 (Table 3). Collectively, the results of the quantitative RT-PCR experiments verified the results of the microarray analysis and indicated that englerin A induced ER stress signaling and an inflammatory response.

**Table 3:** Real time PCR confirms upregulation of genes associated with the unfolded protein response and inflammation.

A498				UO-31	
Gene	Fold Change (microarray/qPCR)			Fold Change (qPCR)	
	3 h	8 h	20 h	3 h	8 h
ATF4	1.9/2.2	1.7/2.2	1.3/1.3	1.5	3.3
EGR1	24.4/31.7	11.3/18.8	2.8/3.7	15.2	9.4
EIF4A2	2.6/2.1	2.0/2.3	1.3/1.8	2.2	2.2
PPP1R15A	7.8/9.6	7.6/11.3	3.4/4.5	6.0	8.3
TNFα	15.6/23.4	22.8/56.7	10.5/18.6	1.0	14.3
IL-6	9.5/12.2	5.4/8.7	3.1/5.1	0.8	8.8
CXCL2	10.5/13.6	8.2/11.3	6.5/7.9	0.8	2.5

Reverse transcription and real time PCR was conducted for select genes using RNA isolated from A498 and UO-31 renal carcinoma cells treated with 0.1% DMSO (vehicle) or englerin A at 40 nM (UO-31) or 100 nM for the indicated times. The fold change in select genes obtained from both microarray and real time PCR experiments are presented as indicated.

**Fig 2:**
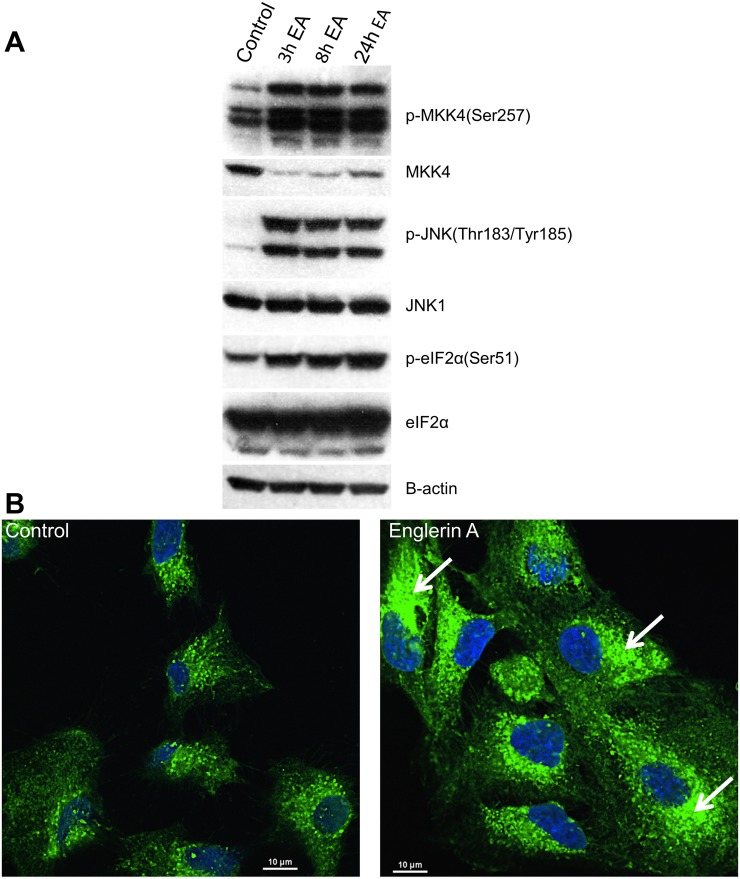
Englerin A induces ER stress signaling and morphological changes in the ER. Western blot analysis (A) was performed using protein extracts obtained from A498 cells treated with 0.1% DMSO (vehicle) or 100 nM englerin A for the indicated times. ER morphology (B) was imaged by fluorescence confocal microscopy of UO-31 cells that were treated with vehicle or 25 nM englerin A for 28.5h followed by staining of cells with ER-ID^®^ Green and Hoechst stain. Arrows show areas of the ER with disrupted fine tubular network.

In order to confirm at the phosphoprotein level that englerin A induced an unfolded protein and inflammatory response, Western Blot analysis using phospho-specific antibodies was conducted to examine signaling pathways characteristic of these responses. One of the hallmarks and earliest events following ER stress is the phosphorylation of eIF2α by PERK, the major protein responsible for attenuation of mRNA translation. Western analysis revealed that phosphorylation of eIF2α was induced by englerin A (100 nM) in A498 cells at 3 h with the greatest level of phosphorylation occurring at 24 h (Fig 2A). We also examined the phosphorylation of the stress kinase, JNK, and its upstream kinase MKK4/7 which are activated by the ER stress sensor, inositol requiring enzyme 1, IRE1α. Furthermore, activation of JNK is a key step in the induction of inflammatory cytokines via AP1 [46,51]. Western blot analysis confirmed that both MKK4/7 and JNK were activated by englerin A by 3 h (Fig 2A). Collectively, these findings support results from both microarray and quantitative PCR experiments and confirm that englerin A induces an unfolded protein response as well as an inflammatory response.

### Englerin A disrupts ER morphology

Since englerin A induces ER stress signaling we sought to determine whether englerin A, like some ER stress inducing agents, could disrupt the morphology of the ER further confirming the deleterious effect of englerin A on the ER. In these experiments, UO-31 cells were treated with 0.1% DMSO or englerin A (25 nM) for 28.5 h and then stained with ER-ID^®^ Green and Hoechst stain followed by imaging by fluorescent confocal microscopy. As shown in Fig 2B, control cells treated with 0.1% DMSO display a fine tubular network characteristic of an unstressed ER whereas cells treated with englerin A display disruption of this network and the formation of sack-like cisternae with increased staining typical of a stressed ER [52,53]. Similar results were observed in A498 cells treated with englerin A in separate independent experiments (data not shown). Our finding that englerin A disrupts ER morphology is consistent with our metabolomics results revealing that englerin A generates ceramides and depletes phosphatidylcholine and other phospholipids (at 24 h) as well as with our microarray and real time PCR results demonstrating ER stress induced unfolded protein response signaling. Thus, englerin A not only induces ER stress signaling but also disrupts the normal morphology of the ER.

### Ceramides are toxic to A498 and UO-31 renal carcinoma cells

**Fig 3:**
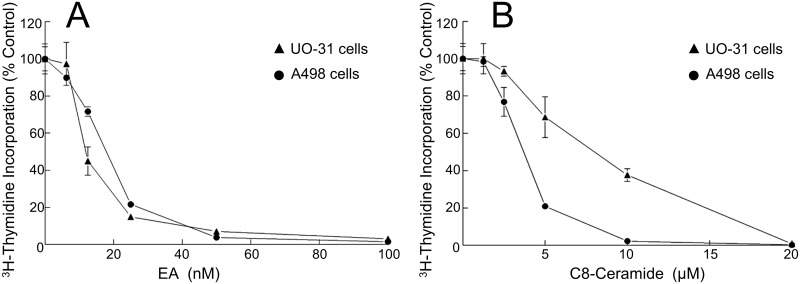
Renal cancer cells are highly sensitive to growth inhibition by englerin A and ceramide. [^3^H]-Thymidine incorporation was measured in A498 and UO-31 cells treated with 0.1% DMSO (control) or increasing concentrations of englerin A (A) or C8-ceramide (B) for 48 h.

The induction of ceramides by different stimuli is known to cause ER stress and cell death [39,43]. In order to confirm that A498 and UO-31 cells which are two of the most sensitive cell lines to the cytotoxicity of englerin A (IC_50_ < 20 nM) (Fig 3A) [18,22] are also sensitive to the cytotoxicity of ceramides, proliferation assays were conducted. For these experiments, A498 and UO-31 cells were treated with increasing concentrations of englerin A or C8-ceramide for 48 h followed by measurement of cell proliferation using [^3^H]-thymidine incorporation. It is important to note that, unlike endogenously induced ceramides, exogenously added ceramides have difficulty reaching their targets as they do not penetrate cells well and are subject to sticking to inanimate surfaces thus necessitating the use of micro molar levels in various assays to observe an effect. As shown in Fig 3B, both A498 and UO-31 cells were sensitive to the cytotoxicity of C8-ceramide with an IC_50_ of 4 μM and 7 μM, respectively. Interestingly, C8-ceramide1-phosphate was also toxic to A498 cells with an IC_50_ less than 7.5 μM (data not shown). On the other hand, C16-ceramide which does not penetrate cells did not have any effect on cell proliferation (data not shown). Our results indicate that both A498 and UO-31 cells are sensitive to the cytotoxicity of ceramides and that ceramides likely mediate some of the effects of englerin A, especially relating to ER stress and inflammation. Attempts to block the generation of ceramides with various inhibitors of acid and neutral sphingomyelinases, the enzymes activated by various stress stimuli to generate ceramides, were uninformative due to the inherent toxicity of these inhibitors to A498 and UO-31 cells (data not shown). Hence, future studies will aim to elucidate the mechanism by which ceramides are generated in response to engerin A and delineate which actions of englerin A ceramides may mediate.

## Discussion

In the current study, we have found that englerin A profoundly altered lipid metabolism in A498 cc-RCC cells and generated a significant level of ceramides. We further found that engerin A induced ER stress/unfolded protein response signaling in both A498 and UO-31 cells. The induction of ER stress was confirmed by confocal microscopy imaging revealing that englerin A altered the normal morphology of the ER. Lastly, our results indicated that englerin A induced a significant inflammatory response with the induction of signaling by interferon α/ß as well as signaling by inflammatory interleukins and TNF.

Though many tumors rely on increased glucose uptake and glycolysis, few accumulate lipids to the extent of cc-RCC, giving it its distinct clear cell phenotype. The presence of extensive lipid droplets in cc-RCC suggests profoundly altered lipid metabolism compared to normal cells. In fact, there is increasing evidence of altered lipid metabolism in cc-RCC including aberrant expression of fatty acid synthase, steroyl-CoA desaturase 1 (SCD1), A:cholesterol acyl transferase, glucosylceramide synthase and several other lipogenic genes [13–15]. These findings suggest that this aberrant lipid metabolism is necessary for the survival of cc-RCC and possibly for other fat storing tumor cells. Therefore, targeting this aberrant lipid metabolism in such cells may be a strategy to selectively kill them. Evidence supporting this notion is mounting and further reveals that perturbations of lipid metabolism in cc-RCC results in ER stress and cell death. For example, one study targeted SCD1, overexpressed in cc-RCC, using a novel inhibitor and showed that inhibition or genetic knockdown of SCD1 resulted in ER stress and apoptosis *in vitro* and *in vivo* [14]. Moreover, a recent study clearly showed a connection between lipid storage, ER homeostasis, and viability in cc-RCC [54]. In particular, this study demonstrated the presence of highly elevated levels of the lipid droplet coat protein, PLIN2, in cc-RCC that functioned to enable lipid storage. In addition, it was found that increased PLIN2 expression and lipid storage were driven by HIF2 alpha and were required to maintain ER homeostasis and cell viability. Suppression of PLIN2 expression resulted in reduced lipid storage, ER stress, and cell death. These studies revealed lipid metabolism and ER stress as a targetable vulnerability in the most common form of RCC. Based on the findings of these and similar studies, it appears that englerin A, by altering lipid metabolism induces ER stress and cell death. However, since the metabolomics experiments in the current study were conducted using A498 cells only, it may not be possible to extrapolate to other cc-RCC cells in regards to the disruption of lipid metabolism by englerin A culminating in ER stress and cell death. Notably, according to the results of cytotoxicity profiling of englerin A in over 500 cancer cell lines, [21] the cancer cell types most sensitive to englerin A, in addition to renal cancer cells, included those of bone, central nervous system, and liver, all of which are known to store relatively large amounts of lipids [55–59]. This finding is in line with our results and further supports lipid metabolism and ER stress as a targetable vulnerability in lipid storing tumor cells.

In altering lipid metabolism, englerin A significantly increases the levels of ceramides, likely by activating sphingomyelinases. We found that ceramides are highly toxic to both cc-RCC cell lines we tested. Ceramides are known to induce ER stress, resulting in the unfolded protein response, as well as participate in autophagic response signaling [39]. We have previously shown that engerin A induced autophagy [22] in contrast to others [19,24] who did not observe autophagy in response to englerin A. However, in these studies, autophagy was likely inhibited by the supplementation of the cell culture media with non-essential amino acids, which is known to act as an inhibitor of autophagy [60]. In this study, we demonstrated that englerin A induced ER stress at the molecular, cellular, and organelle level. Moreover, we have previously found that englerin A inhibited Akt activation, another action common to that of ceramides [22]. Collectively, our results indicate that ceramides and englerin A have several actions in common. Adding to this list of shared activities, a recent study reported that englerin A activated PKCθ, thereby inducing insulin resistance and inhibition of glucose uptake [23]. It is well established that ceramides induce insulin resistance [61–63] as well as inhibit glucose uptake [64]. Moreover, ceramides have been shown to activate the atypical PKC isoform PKCζ [65] and, like diacylglycerol (DAG), activate PKCθ in skeletal muscle [66,67]. Hence, it is possible that englerin A may activate PKCθ via induction of ceramides in addition to direct activation. Another well known activity of ceramides is the regulation of intracellular calcium levels and modulation of calcium channels [68–70]. Englerin A has been established to increase intracellular calcium levels [19] and this activity too may be modulated by ceramides. Though ceramides inhibit the L-type calcium channels [68], as englerin A was recently reported to do [26], it is unlikely that ceramides mediate this effect of englerin A. Modulation of L-type calcium channels by englerin A occurred at micromolar levels (Ki = 5.7 μM) while ceramides accumulate significantly in cc-RCC cells at nanomolar concentrations of englerin A. Ceramides were not found to modulate the TRPC4/5 channels and thus do not mediate englerin A activation of TRPC4/5 channels [71]. However, our collective results including the significant rise in ceramides in A498 cells in response to englerin A, as well as the many activities that ceramides and englerin A have in common, suggest that ceramides may mediate some of the actions of englerin A, especially in inducing ER stress, autophagy, and inflammation.

Our results also indicated that englerin induced an inflammatory response that may in part be due to ER stress. This inflammatory response was an acute inflammatory response characterized by acute phase response signaling, activation of IRF by cytosolic pattern recognition receptors, pathogen induced signaling, and a RIG-I/MDA5 mediated induction of IFN-alpha/beta pathways as determined by Ingenuity and GeneSpring pathway analyses. Though chronic inflammation may promote tumorigenesis, acute inflammation can result in tumor regression. For instance, the agent Calmette-Guerin, an agent that induces an acute inflammatory response [72], has been used for decades to induce tumor regression in bladder cancer patients. Moreover, in one study, the use of inflammatory agents including polyinosinic:polycytidylic, Bacillus Calmette Guerin, Complete Freund’s adjuvant, and Incomplete Freund’s Adjuvant induced anti-tumor activity in CD1 mice injected with Ehrlich ascites carcinoma [73]. The induction of an acute inflammatory response involving interferon signaling is believed to allow priming of T-cells and infiltration of these cells into tumors, thereby providing anti-tumor immunity. A very recent study has demonstrated this concept by using synthetic cyclic dinucleotides to activate stimulator of interferon genes (STING), a transmembrane protein localized to the ER that undergoes a conformational change in response to direct binding of cyclic dinucleotides, resulting in a downstream signaling cascade involving TBK1 activation, IRF-3 phosphorylation, and production of IFN-ß and other cytokines [74]. Intratumoral injection of this STING activator induced regression of established tumors and generated systemic immune responses, mediating rejection of distant metastases and providing immunologic memory. These findings suggest that englerin A, by activating INF-α/ß signaling in addition to that of other inflammatory cytokines, likely by ER stress, may prime T-cells in the tumor microenvironment to result in anti-tumor immunity and tumor regression [75].

## Conclusion

Using a systems biology approach, the current study revealed for the first time that englerin A, at nanomolar concentrations, profoundly disrupted lipid metabolism unique to cc-RCC and generated ceramides that may mediate some of the effects of englerin A. Furthermore, our study revealed that engerin A induced ER stress and an acute inflammatory response akin to responses against pathogens. Importantly, these findings suggest that cc-RCC is highly sensitive to disruptions in lipid metabolism and ER stress and that these vulnerabilities can be targeted for the treatment of cc-RCC and possibly other lipid storing cancers. Lastly, the acute inflammatory response induced by englerin A may mediate anti-tumor immunity.

## Supporting information

S1 Table Top metabolites changed by englerin treatment for 24 h. (PPTX)

S2 Table Top Metabolites Changed by englerin treatment for 48 h. (PPTX)

S3 Table Significantly altered genes by englerin A. (XLSX)

S1 List of abbreviations (DOCX)
