# Supplementary material for: Englerin A induces an acute inflammatory response and reveals lipid metabolism and ER stress as targetable vulnerabilities in renal cell carcinoma
Source: PLoS One. 2017 Mar 15;12(3):e0172632. doi: 10.1371/journal.pone.0172632 (PMC5351975; doi:10.1371/journal.pone.0172632)
Supplement: S1 Table — (PPTX) [file pone.0172632.s001.pptx]

## Slide 1
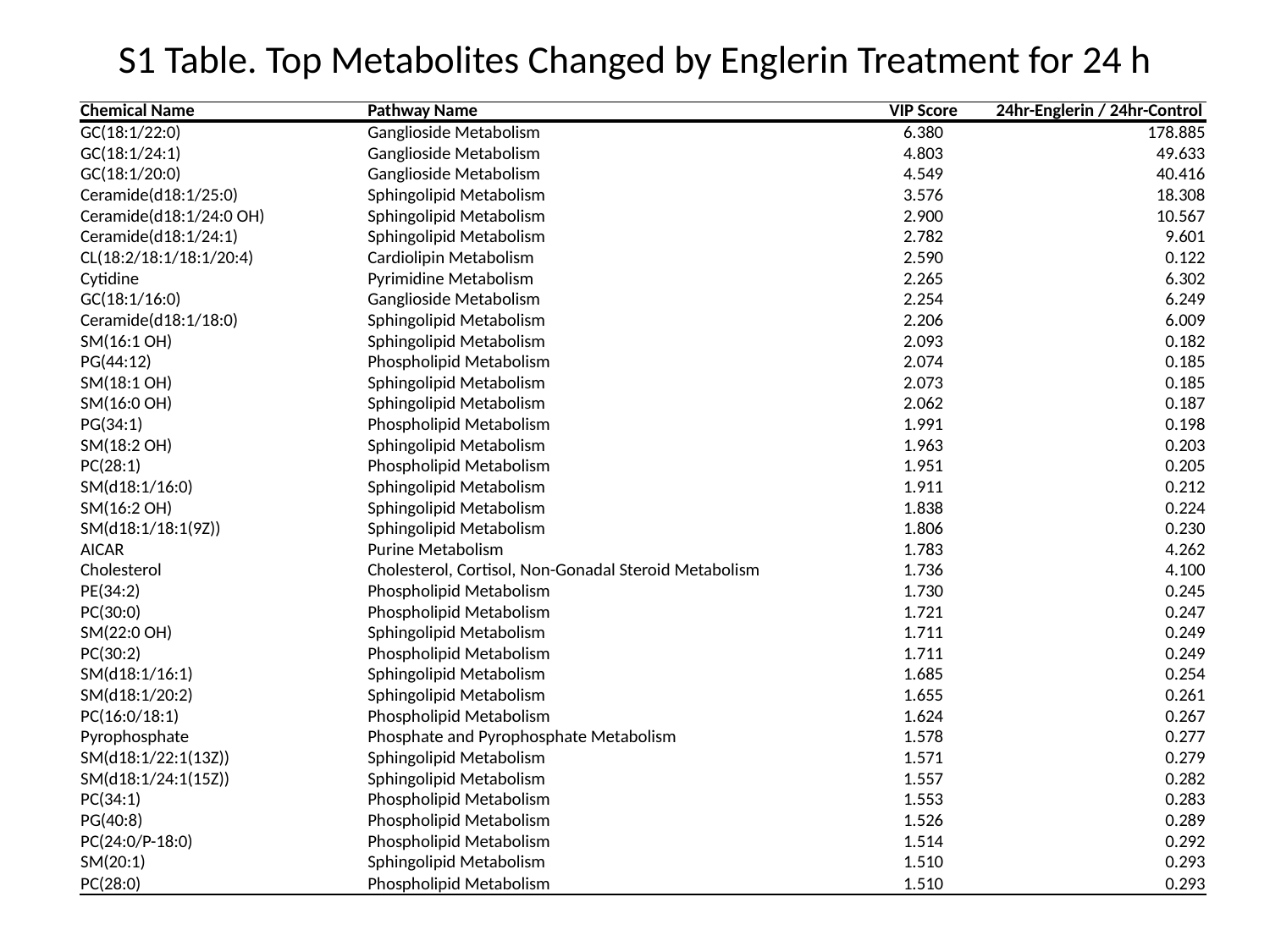

S1 Table. Top Metabolites Changed by Englerin Treatment for 24 h
| Chemical Name | Pathway Name | VIP Score | 24hr-Englerin / 24hr-Control |
| --- | --- | --- | --- |
| GC(18:1/22:0) | Ganglioside Metabolism | 6.380 | 178.885 |
| GC(18:1/24:1) | Ganglioside Metabolism | 4.803 | 49.633 |
| GC(18:1/20:0) | Ganglioside Metabolism | 4.549 | 40.416 |
| Ceramide(d18:1/25:0) | Sphingolipid Metabolism | 3.576 | 18.308 |
| Ceramide(d18:1/24:0 OH) | Sphingolipid Metabolism | 2.900 | 10.567 |
| Ceramide(d18:1/24:1) | Sphingolipid Metabolism | 2.782 | 9.601 |
| CL(18:2/18:1/18:1/20:4) | Cardiolipin Metabolism | 2.590 | 0.122 |
| Cytidine | Pyrimidine Metabolism | 2.265 | 6.302 |
| GC(18:1/16:0) | Ganglioside Metabolism | 2.254 | 6.249 |
| Ceramide(d18:1/18:0) | Sphingolipid Metabolism | 2.206 | 6.009 |
| SM(16:1 OH) | Sphingolipid Metabolism | 2.093 | 0.182 |
| PG(44:12) | Phospholipid Metabolism | 2.074 | 0.185 |
| SM(18:1 OH) | Sphingolipid Metabolism | 2.073 | 0.185 |
| SM(16:0 OH) | Sphingolipid Metabolism | 2.062 | 0.187 |
| PG(34:1) | Phospholipid Metabolism | 1.991 | 0.198 |
| SM(18:2 OH) | Sphingolipid Metabolism | 1.963 | 0.203 |
| PC(28:1) | Phospholipid Metabolism | 1.951 | 0.205 |
| SM(d18:1/16:0) | Sphingolipid Metabolism | 1.911 | 0.212 |
| SM(16:2 OH) | Sphingolipid Metabolism | 1.838 | 0.224 |
| SM(d18:1/18:1(9Z)) | Sphingolipid Metabolism | 1.806 | 0.230 |
| AICAR | Purine Metabolism | 1.783 | 4.262 |
| Cholesterol | Cholesterol, Cortisol, Non-Gonadal Steroid Metabolism | 1.736 | 4.100 |
| PE(34:2) | Phospholipid Metabolism | 1.730 | 0.245 |
| PC(30:0) | Phospholipid Metabolism | 1.721 | 0.247 |
| SM(22:0 OH) | Sphingolipid Metabolism | 1.711 | 0.249 |
| PC(30:2) | Phospholipid Metabolism | 1.711 | 0.249 |
| SM(d18:1/16:1) | Sphingolipid Metabolism | 1.685 | 0.254 |
| SM(d18:1/20:2) | Sphingolipid Metabolism | 1.655 | 0.261 |
| PC(16:0/18:1) | Phospholipid Metabolism | 1.624 | 0.267 |
| Pyrophosphate | Phosphate and Pyrophosphate Metabolism | 1.578 | 0.277 |
| SM(d18:1/22:1(13Z)) | Sphingolipid Metabolism | 1.571 | 0.279 |
| SM(d18:1/24:1(15Z)) | Sphingolipid Metabolism | 1.557 | 0.282 |
| PC(34:1) | Phospholipid Metabolism | 1.553 | 0.283 |
| PG(40:8) | Phospholipid Metabolism | 1.526 | 0.289 |
| PC(24:0/P-18:0) | Phospholipid Metabolism | 1.514 | 0.292 |
| SM(20:1) | Sphingolipid Metabolism | 1.510 | 0.293 |
| PC(28:0) | Phospholipid Metabolism | 1.510 | 0.293 |
