# Supplementary material for: Englerin A induces an acute inflammatory response and reveals lipid metabolism and ER stress as targetable vulnerabilities in renal cell carcinoma
Source: PLoS One. 2017 Mar 15;12(3):e0172632. doi: 10.1371/journal.pone.0172632 (PMC5351975; doi:10.1371/journal.pone.0172632)
Supplement: S2 Table — (PPTX) [file pone.0172632.s002.pptx]

## Slide 1
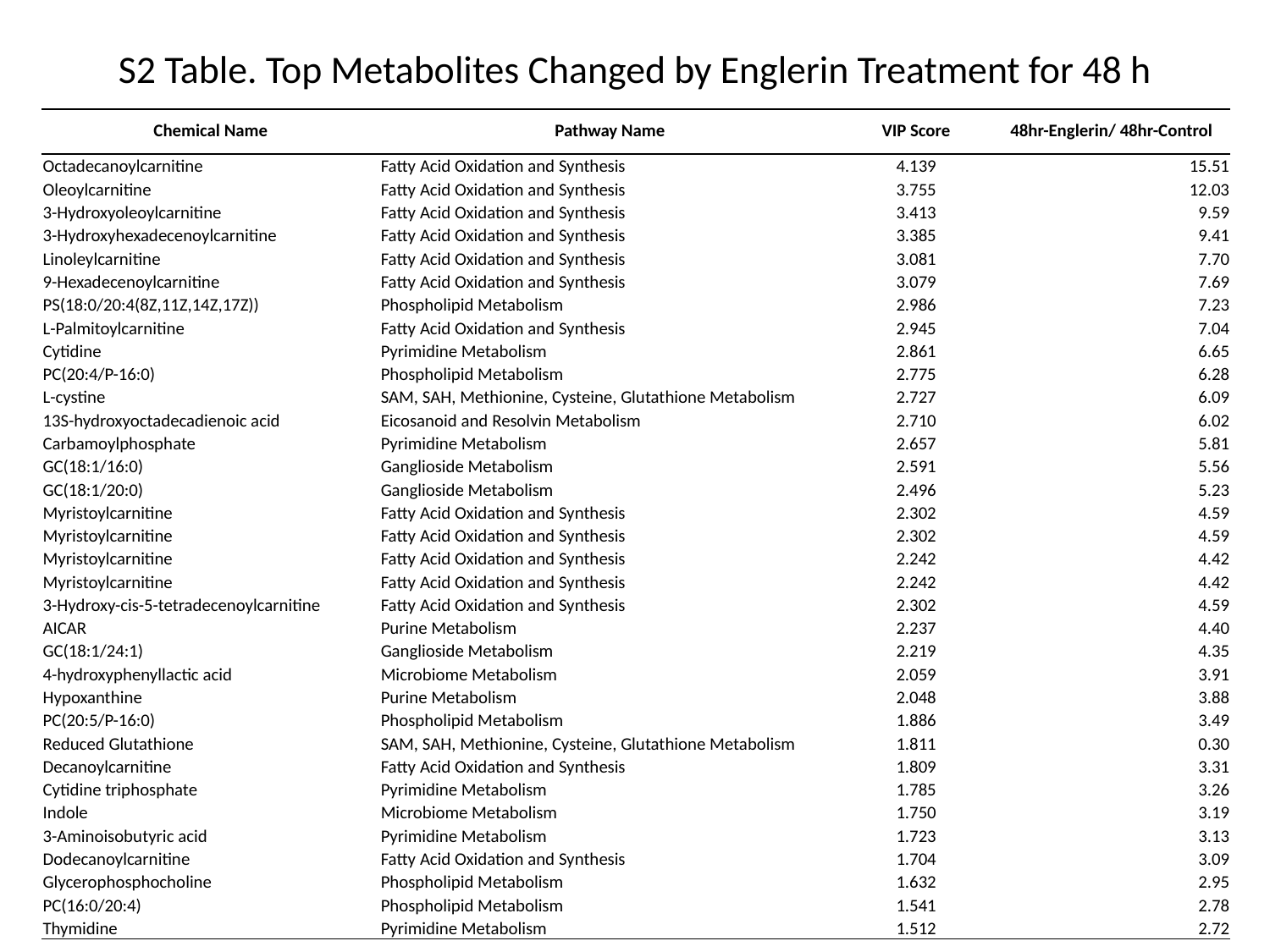

S2 Table. Top Metabolites Changed by Englerin Treatment for 48 h
| Chemical Name | Pathway Name | VIP Score | 48hr-Englerin/ 48hr-Control |
| --- | --- | --- | --- |
| Octadecanoylcarnitine | Fatty Acid Oxidation and Synthesis | 4.139 | 15.51 |
| Oleoylcarnitine | Fatty Acid Oxidation and Synthesis | 3.755 | 12.03 |
| 3-Hydroxyoleoylcarnitine | Fatty Acid Oxidation and Synthesis | 3.413 | 9.59 |
| 3-Hydroxyhexadecenoylcarnitine | Fatty Acid Oxidation and Synthesis | 3.385 | 9.41 |
| Linoleylcarnitine | Fatty Acid Oxidation and Synthesis | 3.081 | 7.70 |
| 9-Hexadecenoylcarnitine | Fatty Acid Oxidation and Synthesis | 3.079 | 7.69 |
| PS(18:0/20:4(8Z,11Z,14Z,17Z)) | Phospholipid Metabolism | 2.986 | 7.23 |
| L-Palmitoylcarnitine | Fatty Acid Oxidation and Synthesis | 2.945 | 7.04 |
| Cytidine | Pyrimidine Metabolism | 2.861 | 6.65 |
| PC(20:4/P-16:0) | Phospholipid Metabolism | 2.775 | 6.28 |
| L-cystine | SAM, SAH, Methionine, Cysteine, Glutathione Metabolism | 2.727 | 6.09 |
| 13S-hydroxyoctadecadienoic acid | Eicosanoid and Resolvin Metabolism | 2.710 | 6.02 |
| Carbamoylphosphate | Pyrimidine Metabolism | 2.657 | 5.81 |
| GC(18:1/16:0) | Ganglioside Metabolism | 2.591 | 5.56 |
| GC(18:1/20:0) | Ganglioside Metabolism | 2.496 | 5.23 |
| Myristoylcarnitine | Fatty Acid Oxidation and Synthesis | 2.302 | 4.59 |
| Myristoylcarnitine | Fatty Acid Oxidation and Synthesis | 2.302 | 4.59 |
| Myristoylcarnitine | Fatty Acid Oxidation and Synthesis | 2.242 | 4.42 |
| Myristoylcarnitine | Fatty Acid Oxidation and Synthesis | 2.242 | 4.42 |
| 3-Hydroxy-cis-5-tetradecenoylcarnitine | Fatty Acid Oxidation and Synthesis | 2.302 | 4.59 |
| AICAR | Purine Metabolism | 2.237 | 4.40 |
| GC(18:1/24:1) | Ganglioside Metabolism | 2.219 | 4.35 |
| 4-hydroxyphenyllactic acid | Microbiome Metabolism | 2.059 | 3.91 |
| Hypoxanthine | Purine Metabolism | 2.048 | 3.88 |
| PC(20:5/P-16:0) | Phospholipid Metabolism | 1.886 | 3.49 |
| Reduced Glutathione | SAM, SAH, Methionine, Cysteine, Glutathione Metabolism | 1.811 | 0.30 |
| Decanoylcarnitine | Fatty Acid Oxidation and Synthesis | 1.809 | 3.31 |
| Cytidine triphosphate | Pyrimidine Metabolism | 1.785 | 3.26 |
| Indole | Microbiome Metabolism | 1.750 | 3.19 |
| 3-Aminoisobutyric acid | Pyrimidine Metabolism | 1.723 | 3.13 |
| Dodecanoylcarnitine | Fatty Acid Oxidation and Synthesis | 1.704 | 3.09 |
| Glycerophosphocholine | Phospholipid Metabolism | 1.632 | 2.95 |
| PC(16:0/20:4) | Phospholipid Metabolism | 1.541 | 2.78 |
| Thymidine | Pyrimidine Metabolism | 1.512 | 2.72 |
