# Supplementary material for: Englerin A induces an acute inflammatory response and reveals lipid metabolism and ER stress as targetable vulnerabilities in renal cell carcinoma
Source: PLoS One. 2017 Mar 15;12(3):e0172632. doi: 10.1371/journal.pone.0172632 (PMC5351975; doi:10.1371/journal.pone.0172632)
Supplement: S1 List of abbreviations — (DOCX) [file pone.0172632.s004.docx]

**List of Abbreviations**

AICAR 5-Aminoimidazole-4-carboxamide ribonucleotide

ATCC American type culture collection

ATF Activating Transcription Factor

CCL Chemokine (C-C motif) ligand

CHOP C/EBP homologous protein

CL Cardiolipin

CXCL Chemokine (C-X-C motif) ligand

DMSO Dimethyl sulfoxide

EGR1 Early growth response 1

EIF Eukaryotic Translation Initiation Factor

ER Endoplasmic reticulum

Erk Extracellular Signal-regulated Kinase

ERB Estrogen receptor

EWS-FLI1 Ewing’s sarcoma-friend leukemia integration 1 transcription factor

FBS Fetal bovine serum

GADD34 Growth Arrest And DNA Damage-Inducible Protein

GC Glucosylceramide

HIF2 Hypoxia inducible factor 2

IFN interferon

IL Interleukin

IRF Interferon regulatory factor

JNK c-Jun N-terminal kinase

MAPK Mitogen-activated protein kinase

MDA5 Melanoma Differentiation-Associated protein 5

MKK4 Mitogen-activated protein kinase kinase 4

NFKB Nuclear factor Kappa B

PC phosphatidylcholine

PDGF Platelet derived growth factor

PE phosphatidylethanolamine

PERK Protein kinase R-like endoplasmic reticulum kinase

PKC Protein Kinase C

PLIN2 Perilipin 2

PS phosphatidylserine

RIG-1 Retinoic acid-inducible gene 1

RPMI Roswell Park Memorial Institute medium

SM Sphingomyelin

TBK1 TANK Binding Kinase 1

TGF-B Transforming growth factor Beta

TNF Tumor necrosis factor
